# Plasma B-type Natriuretic Peptide as a Predictor of Cardiovascular Events in Subjects with Atrial Fibrillation: A Community-Based Study

**DOI:** 10.1371/journal.pone.0081243

**Published:** 2013-12-02

**Authors:** Motoyuki Nakamura, Yorihiko Koeda, Fumitaka Tanaka, Toshiyuki Onoda, Kazuyoshi Itai, Masaki Ohsawa, Kozo Tanno, Kiyomi Sakata, Shinich Omama, Yasuhiro Ishibashi, Shinji Makita, Mutsuko Ohta, Kuniaki Ogasawara, Takashi Komatsu, Akira Okayama

**Affiliations:** 1 Department of Internal Medicine, Iwate Medical University, Morioka, Japan; 2 Department of Hygiene and Preventive Medicine, Iwate Medical University, Morioka, Japan; 3 Department of Neurosurgery, Iwate Medical University, Morioka, Japan; 4 Iwate Health Service Association, Morioka, Japan; 5 The First Institute of Health Service, Japan Anti-Tuberculosis Association, Tokyo, Japan; University Hospital Medical Centre, Germany

## Abstract

**Objectives:**

Atrial fibrillation (AF) is a significant public health issue due to its high prevalence in the general population, and is associated with an increased risk of cardiovascular (CV) events including systemic thrombo-embolism, heart failure, and coronary artery disease. The relationship between plasma B-type natriuretic peptide (BNP) and CV risk in real world AF subjects remains unknown.

**Methods:**

The subject of the study (n = 228; mean age = 69 years) was unselected individuals with AF in a community-based population (n = 15,394; AF prevalence rate = 1.5%). The CV event free rate within each BNP tertile was estimated, and Cox regression analysis was performed to examine the relative risk of the onset of CV events among the tertiles. The prognostic ability of BNP was compared to an established risk score for embolic events (CHADS2 score). In addition, to determine the usefulness of BNP as a predictor in addition to CHADS2 score, we calculated Net Reclassification Improvement (NRI) and Integrated Discrimination Improvement (IDI) indices.

**Results:**

During the follow-up period 58 subjects experienced CV events (52 per 1,000 person-years). The event-free ratio was significantly lower in the highest tertile (p < 0.02). After adjustment for established CV risk factors, the hazard ratio (HR) of the highest tertile was significantly higher than that of the lowest tertile (HR = 2.38; p < 0.02). The predictive abilities of plasma BNP in terms of sensitivity and specificity for general CV events were comparable to those of CHADS2 score. Adding BNP to the CHADS2 score only model improved the NRI (0.319; p < 0.05) and the IDI (0.046; p < 0.05).

**Conclusion:**

Plasma BNP is a valuable biomarker both singly or in combination with an established scoring system for assessing general CV risk including stroke, heart failure and acute coronary syndrome in real-world AF subjects.

## Introduction

Atrial fibrillation (AF) is a significant public health issue due to its high prevalence in the general population, and is associated with an increased risk of cardiovascular (CV) events including systemic thrombo-embolism, heart failure, and coronary artery disease [1-3]. In recent years, several types of scoring systems for predicting risk of embolic events have been developed [4,5]. However, AF is a risk factor not only for systemic thrombo-embolism but also for development of heart failure and coronary heart disease [1-3,6,7]. There are no simple established biomarkers to stratify general CV risk in AF patients. Increased circulating levels of B-type natriuretic peptide (BNP) and its N-terminal fragment (NT-pro BNP) have been reported to be related to high risk of cardiovascular events and mortality [8-10]. In patients with AF, plasma BNP levels were significantly higher than in control subjects with sinus rhythm [11,12]. However, it remains unclear whether plasma BNP levels might be a reliable biomarker for prediction of general CV events, including stroke, heart failure and coronary heart disease, in AF cohorts selected from community-based populations. 

## Methods

### Study population

The original cohort of the Iwate-KENCO study was recruited from a community-based population living in Ninohe, Kuji, and Miyako districts of the northern Iwate prefecture, Japan. Baseline examinations including BNP measurement and ECG recording were performed between 2002 and 2004. Details of recruitment and baseline measurements have been described in previous reports [13,14]. All of the subjects used a self-report questionnaire to confirm medical history including the status (yes or no) of prescribed drugs for hypertension, diabetes, hypercholesterolemia, stroke, angina, heart failure and myocardial infarction. The smoking status (current, past, or non smoker) was also assessed by a questionnaire. The total number of participants who agreed to join the Iwate-KENCO study in the three districts was 26,469 (original cohort). Of the original cohort living in the Ninohe and Kuji districts (n = 15,927), 15,394 subjects (97%) had BNP measurements (BNP cohort: men 5,288; women 10,106). Among this BNP cohort, the AF subjects were selected on the basis of standard 12-lead ECG tracings obtained at baseline examination. The diagnosis of AF was established by the ECG recorder machine-inherent algorithm, and the ECG tracing was examined by laboratory technologists and confirmed by expert medical doctors. In the present study, the subjects with atrial flutter were designated as the AF cohort. Subjects having the following factors were excluded from the study: age under 40 years; estimated GFR below 30 ml/min/1.73m^2^; missing data for the covariates. The final number of subjects with AF was 228 (male 170, female 58; mean age 69.3: Table 1). The study protocol (#H13-33) was approved by the ethics committee (Iwate Medical University Institutional Review Board #1). All of the participants gave written informed consent.

**Table 1:** Clinical characteristics of subjects with AF among tertile of plasma BNP levels.

	**Total (n = 228)**	**T1 (n = 75)**	**T2 (n = 77)**	**T3 (n = 76)**	*p* values*
**Median BNP pg/ml**	**111**	**49**	**111**	**200**	
**(IQR)**	**(67 - 170)**	**(26 - 67)**	**(92 - 121)**	**(169 - 243)**	
**Age (years)**	**69.3 ± 7.9**	**66.2 ± 9.1**	**69.1 ± 7.5**	**72.2 ± 5.8**	**0.001**
**Male**	**74.6%**	**77.3%**	**75.3%**	**71.1%**	**0.663**
**BMI (kg/m^2^)**	**24.6 ± 3.1**	**25.1 ± 3.0**	**24.0 ± 3.0**	**24.6 ± 3.2**	**0.073**
**Hypertension**	**54.8%**	**48.0%**	**53.2%**	**63.2%**	**0.164**
**Hypercholesterolemia**	**9.6%**	**17.3%**	**5.2%**	**6.6%**	**0.022**
**Diabetes**	**13.6%**	**14.7%**	**13.0%**	**13.2%**	**0.947**
**Current smoking**	**13.2%**	**18.7%**	**10.4%**	**10.5%**	**0.227**
**CV history**	**15.8%**	**12.0%**	**20.8%**	**14.5%**	**0.309**
**CV drugs**	**53.5%**	**43.5%**	**58.4%**	**56.6%**	**0.217**
**eGFR (ml/min/1.73m^2^)**	**67.1 ± 13.6**	**70.8 ± 15.5**	**65.9 ± 11.0**	**64.5 ± 13.4**	**0.012**
**CHADS2 score (0 / 1 / 2-)**	**62 / 96/ 70**	**26 / 32 / 17**	**20 / 31 / 26**	**16 / 33 / 27**	**0.131**

* *p* among tertiles

BMI = body mass index, CV = cardiovascular, eGFR = estimated glomerular filtration rate, IQR = interquartile range, T = tertile

### BNP measurement

Plasma BNP concentration was measured by direct radioimmunoassay using monoclonal antibodies specific for human BNP (Shiono RIA BNP kit, Shionogi). Blood samples were drawn from a peripheral vein in the seated position and then centrifuged at 1,500 g for 10 minutes. After separation, the plasma samples were stored frozen at –20°C until transportation to the Shionogi central laboratory for the assay (Osaka, Japan). Cross-reactivity of the antibody was 100% for human BNP and 0.001% for human atrial natriuretic peptide. The intra- and inter-assay coefficients of variation were 5% and 6%, respectively.

### Risk factor definitions

All of the subjects were seated for at least 5 minutes before blood pressure and pulse rate measurement using an automatic device (BP-103i II, model 513000, Nippon Colin). The measurement was performed twice, with the mean value used for the statistical analysis. Hypertension was defined as systolic blood pressure ≥ 140 mm Hg, diastolic blood pressure ≥ 90 mm Hg and/or the use of antihypertensive medication. The body mass index was calculated as weight (kg) divided by the square of height (m^2^). Diabetes was ascertained by the detection of a non-fasting glucose concentration ≥ 200 mg/dl, HbA1c value ≥ 6.5 % and/or the use of anti-diabetic agents including insulin. Hypercholesterolemia was defined as a serum concentration ≥ 240 mg/dl and/or the use of anti-lipidemic medications.

### Outcome

The endpoint of the study was a composite outcome comprising stroke, heart failure, acute myocardial infarction, and sudden cardiac death. Stroke was identified from local stroke registry data at all regional hospitals, and was defined as the sudden onset of a focal neurological deficit of >= 24 hours’ duration and confirmed by brain computed tomography or magnetic resonance imaging [15]. Admission cases of heart failure were confirmed by registration survey data, which records primary hospital discharge diagnosis in all general hospitals located in the study area. Cases of heart failure were defined by the Framingham criteria [16]. Incidence of myocardial infarction was also based on hospital registration survey data. The diagnosis of acute myocardial infarction was based on the MONICA criteria [17]. Based on death certificates, sudden unexpected death within 24 hours after the onset of acute illness was determined by a committee consisted with cardiologists, neurologists, and epidemiologists. When disagreements were found between the documents, the judgment team discussed and agreed upon the appropriate diagnosis. A follow-up survey assessing mortality and migration was carried out after the baseline study. All deaths and migration were confirmed by the official resident registration data issued by the local government offices.

### Statistical analysis

Continuous variables are shown as mean ± SD. AF subjects were divided into tertiles according to their baseline plasma BNP levels. To compare results among tertiles, ANOVA or the Chi-squared test was used as appropriate. The association between the baseline plasma BNP tertile and end point was evaluated. Survival from entry into the study was estimated using the Kaplan-Meier method, followed by a trend test (Log rank). Using a Cox proportional hazards regression model, the hazard ratios (HRs) for the plasma BNP tertiles versus the CV events were assessed. In this multivariable proportional-hazards regression model, adjustments were made for sex and age (model 1), and further adjustments included the body mass index, estimated glomerular filtration rate, hemoglobin, heart rate and presence or absence of hypertension, diabetes, hypercholesterolemia, current smoking, use of antihypertensive drugs (yes or no), and CV history (model 2). For the analyses of CV incidence, person-years were determined at the date of the CV events, date of emigration from the study area, date of death, or end of the follow-up period, whichever came first. The follow-up survey was carried out after the baseline study through to March 2009. Migrations were confirmed by official resident registration data issued by the local government offices (October 2009). 

To compare BNP and the clinical thrombo-embolic scoring systems (CHADS2 and CAHDS2-VASc) in terms of the overall diagnostic accuracy for CV events, receiver operating characteristic (ROC) curves were constructed, and the area under the curve (AUC) and 95% confidence intervals (CI) for each ROC curve were calculated. In addition, to determine the usefulness of plasma BNP as a predictor of CV events in addition to CHADS2 score, we used Net Reclassification Improvement (NRI) and Integrated Discrimination Improvement (IDI) indices [18]. These statistical analyses were performed using SPSS software (version 11.0.1J, Chicago, IL, USA) or R (version 3.0.1). A significant difference was defined as p < 0.05.

## Results

As shown in Table 1, the number of AF cases was 228. The prevalence of AF within the community-based population was 1.5%. Mean age was 69.3 years old, and median BNP level was 111 (IQR 67 - 170) pg/ml. In the non-AF subjects of the whole BNP cohort, the median BNP level was 16 (IQR 8 - 29) pg/ml. In this AF cohort, the prevalence of hypertension, hypercholesterolemia, diabetes, and current smoking were 55%, 10%, 14% and 13%, respectively. The prevalence of CV history was 16%, and approximately 46% of the AF cohort did not declare any CV treatment. Among the BNP tertiles, the mean age differed and increased with levels of BNP (p < 0.001). The percentage of cases receiving CV drugs did not differ significantly among the tertiles (p = 0.217).

**Figure 1:**
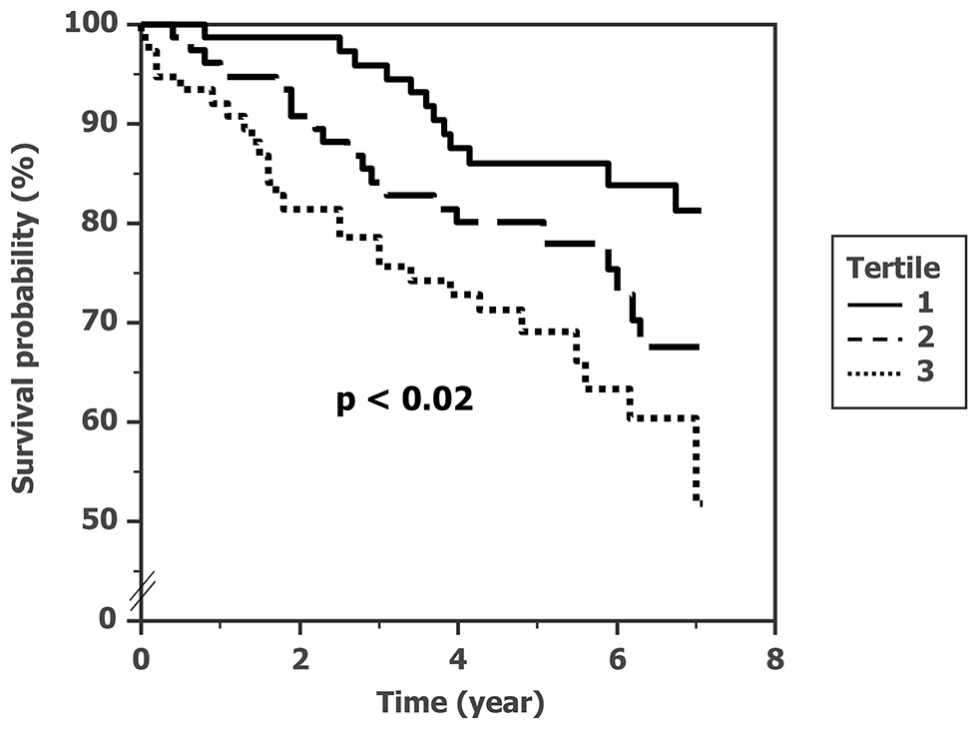
Event-free ratio among the tertiles of plasma BNP in subjects with AF.

The cohort was followed for 1117 person-years. Composite CV events during the follow-up period (median, 5.1 years) were found in 58 cases in the AF cohort. The number of general CV events per 1000 person-years was 52. The Kaplan-Meyer curves for CV event-free rate according to tertile levels of BNP are shown in Figure 1. The CV event-free rate was significantly lower in the highest BNP tertile (*p* for trend < 0.02 by log-rank test). 

**Figure 2:**
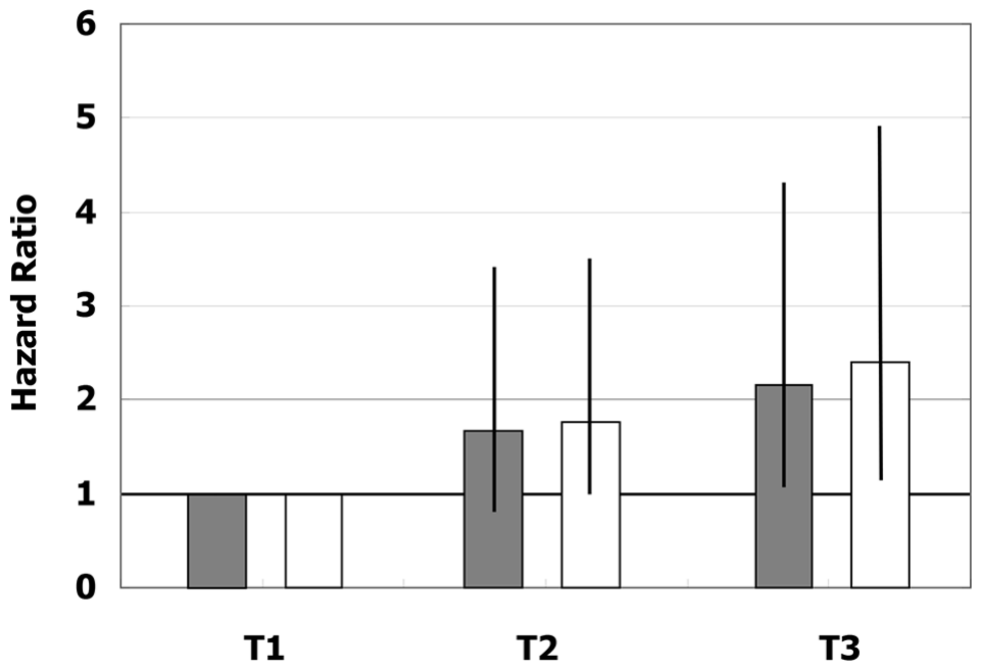
Hazard ratios of Cox regression models among the tertiles of plasma BNP in subjects with AF. Grey bars = age-sex adjusted model; White bars = multivariate model .

Cox regression analysis after the adjustment for age and sex was performed to analyze the relationship between plasma BNP tertiles and the risk of CV events in the AF cohort (model 1). The HR for the highest tertile of plasma BNP was significantly higher than that for the lowest tertile (HR = 2.15; 95% CI = 1.07 to 4.32; p = 0.031: Figure 2). In a multivariate adjustment model (model 2), the relationship between the plasma BNP tertiles and CV event rate was robust (HR = 2.38; 95% CI = 1.15 to 4.93; p = 0.019: Figure 2).

**Figure 3:**
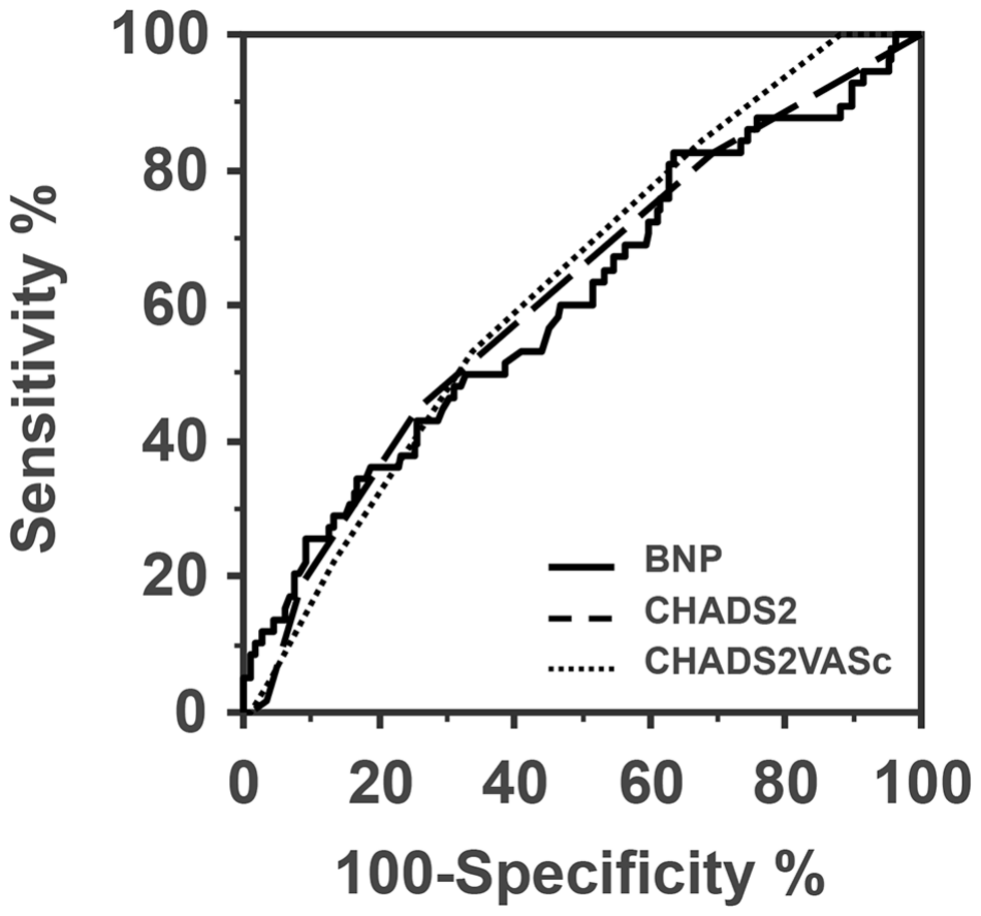
Comparison of ROC curves for prediction of CV events between BNP, CHADS2 and CHADS2-VASc in subjects with AF.

Although the predictive abilities for CV events of the plasma BNP and CHADS2 score was suboptimal (AUC < 0.75), the AUCs of the ROC analysis for prediction of composite CV events were 0.61 (95% CI, 0.54 to 0.67) for BNP and 0.62 (95% CI, 0.55 to 0.68) for the CHADS2 score. The ability to predict the general CV events was comparable between the two markers (p = 0.86: Figure 3). When CHADS2-VASc score used instead for CHADS2 score, the AUC of CHADS2-VASc score (0.63, 95%CI, 0.56 to 0.69) was not significantly different from that of BNP (p = 0.68).

**Figure 4:**
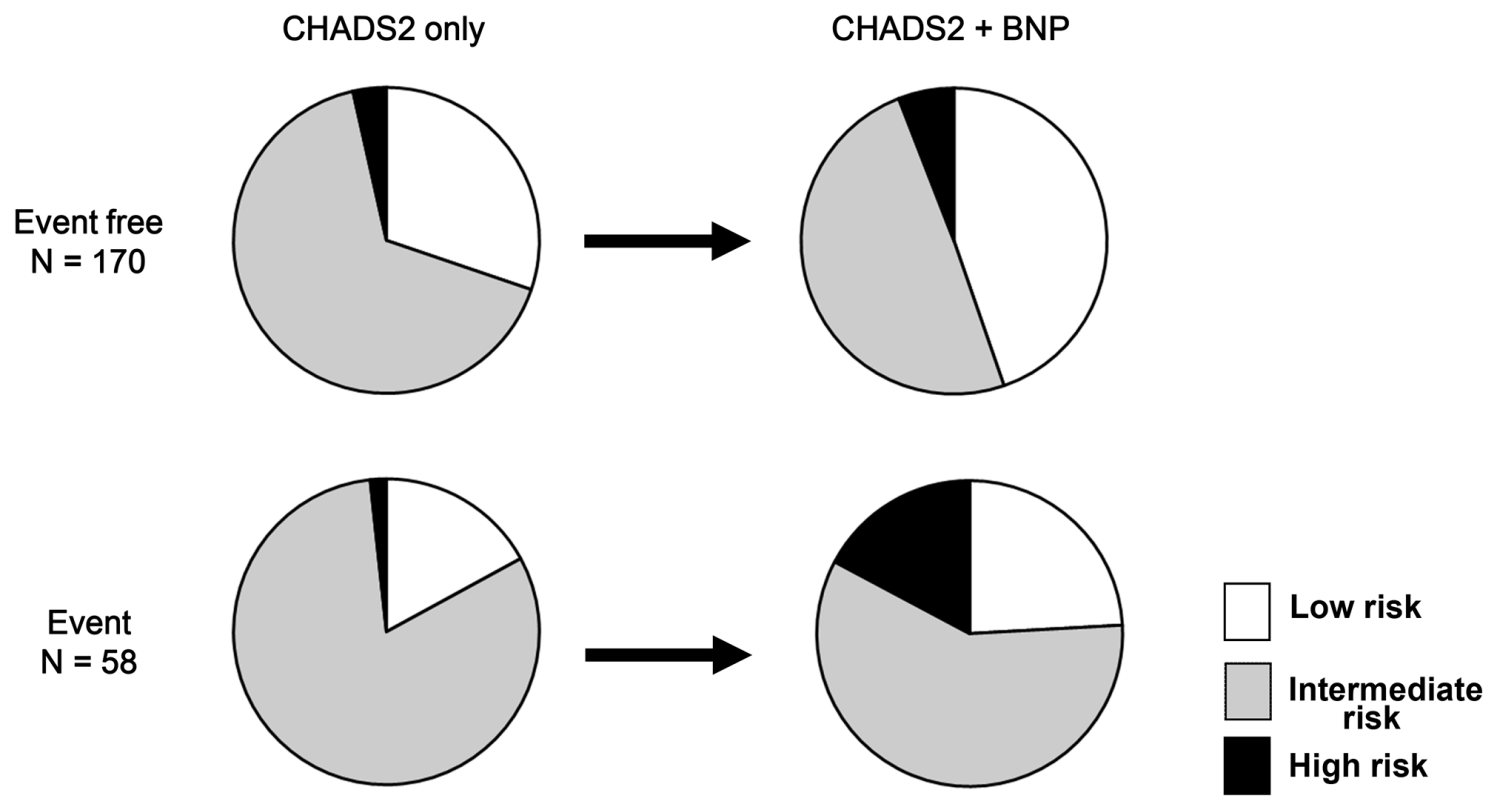
Changes in risk stratification capacity derived from reclassification tables in terms of CHADS2 score only model and CHADS2 plus BNP model. To compare with CHADS2 only model, the combination model increased the percentage of low risk group in the non events group (from 30% to 45%), and the percentage of high risk group in the events group (from 2% to 17%).

The risk stratification capacities of the CV event (eg; low risk < 18%, intermediate risk; 18 - 24%, high risk >= 24%) of the CHADS2 only model and the CHADS2 plus BNP model derived from the reclassification table are shown Figure 4. In the non events group (n = 170), 30% were categorized into the low risk group by the CHADS2 only model, compared to 45% by the CHADS2 plus BNP model. In contrast, in the events group (n = 58), 2% were categorized into the high risk group by CHADS2 only model, with 24% for the CHADS2 plus BNP model. These results indicate that when BNP was added to the CHADS2 model, predictive ability for general CV events was significantly improved (NRI = 0.265, 95% CI; 0.07 - 0.463, p < 0.01: IDI = 0.045, 95% CI; 0.007 - 0.085, p < 0.05).

## Discussion

The present study has demonstrated that plasma BNP is a valuable marker for stratifying general CV risk in unselected subjects with AF. BNP’s usefulness was comparable to that of an established clinical scoring system for thrombo-embolic risk, and further improved this system’s ability for prediction of CV risk.

 BNP is a peptide hormone secreted from the cardiomyocytes mainly in response to increased cardiac wall stress due to increased intracardiac volume and pressure. Several previous studies demonstrated the elevated blood levels of BNP in patients with AF to compare with subjects with normal sinus rhythm (11,12). Plasma BNP levels in patients with AF were significantly correlated with age, left atrial diameter, left ventricular ejection fraction, diastolic functional parameters including left ventricular hypertrophy, and complication of structural heart disease or heart failure [19,20]. Some of these factors are important components of clinical risk stratification scoring systems for thrombo-embolic events in this disorder [5,6]. Given the overlap (age, left ventricular hypertrophy and heart failure) between the promoting factors for natriuretic peptide secretion and embolic risk, the observed relationship between plasma BNP levels and clinical thrombo-embolic risk scores as well as stroke events is easy to understand. In addition, several studies using Doppler echocardiography have reported that reduced left appendage blood flow with enlarged atrial diameter was inversely correlated with the prothrombotic state (high plasma concentrations of D-dimer and thrombin-antithrombin III complex) and plasma BNP in patients with AF [21,22]. These observations suggested that an elevation in the plasma BNP levels is reflected by reduced atrial hemostasis and hypercoagulation states. Therefore, in the present AF cohort, plasma BNP levels may indicate a high risk for intra-atrial thrombus formation, and thus BNP levels are positively related with the stroke event. In fact, two recent substudies of RE-LY and ARISTOTLE demonstrated that plasma NT-proBNP is independently associated with an increased risk of stroke in highly selected AF patients being administered oral anticoagulants [23,24]. These two observations may be accord with our results, however, it is argued whether these findings could extend to real world AF patients with relatively low thrombo-embolic risk such as CHADS2 and CHADS2-VASc scores (</ = 1) and with various anticoagulated levels [25]. The present study might show these issues in a community-based ‘real world’ AF cohort, and could extend the usefulness of the biomarker as a general CV event predictor.

 The present study has shown that the plasma BNP levels were related not only with the risk of stroke but also with the risk of the development of general CV events including stroke, heart failure, and sudden death. Plasma BNP is well known to be increased with the severity of heart failure, and increased plasma BNP is a prognostic marker in patients with heart failure [26,27]. In the general population, Wang et al. showed that an increment in the plasma BNP and elevated plasma BNP above the 80th percentile in the Framingham cohort was associated with a significant increase in the risk of the new onset of heart failure [8]. In addition, the predictive abilities of the plasma BNP levels for the onset of congestive heart failure have been reported to be optimal in men and women of the general population [10]. These previous studies have suggested that the plasma BNP levels may be a possible screening tool in subjects at high risk for heart failure within the general population. 

However, the utility of plasma BNP measurement for predicting heart failure risk has not been established in patients with this arrhythmia. Many of the present subjects with AF may have inherent preclinical cardiac disorders characterized by borderline abnormalities in intracardiac pressure, left ventricular function, valvular competence, and myocardial circulation. The plasma BNP levels in subjects with subclinical structural heart diseases was reported to be higher than in those without these cardiac abnormalities [28]. As the original cohort of the present study includes apparently healthy subjects who had attended a multi-phasic health checkup, few patients with obvious heart failure were included in the study subjects. We therefore speculate that the elevated levels of plasma BNP in the AF cohort denote latent structural heart diseases such as subclinical cardiac dysfunction, including mildly elevated intracardiac pressure and volume, and myocardial ischemia, thus these individuals are prone to be at risk for heart failure and coronary heart disease.

The present study had several limitations. First, although the cohort may be representative of the real-world situation of AF, the prevalence of anticoagulant medication use in the AF cohort and its control levels in each patient were not known. As the baseline survey was performed in the early 2000s, the usefulness of anticoagulant therapy for lone type AF, especially in individuals with low CV risk, has not been established and has not appeared in any therapeutic guidelines. Therefore, whether the present results extend to AF patients under strict anti-thrombotic therapies is uncertain. Second, as ECG recording was performed only once, patients with paroxysmal AF might have been excluded. Third, the present study did not assess the effect of CV drugs, such as renin-angiotensin inhibitors and diuretics, which might affect the plasma BNP levels and the incidence of the outcomes. 

In conclusion, plasma BNP is a useful biomarker when used singly or in combination with established scoring systems to stratify general CV events, including embolic events, heart failure and acute coronary syndrome, in subjects with AF.
